# Current Landscape of Antiviral Drug Discovery

**DOI:** 10.12688/f1000research.7665.1

**Published:** 2016-02-22

**Authors:** Wade Blair, Christopher Cox

**Affiliations:** 1 Department of Antiviral Research, Merck Research Laboratories, West Point, Pennsylvania, 19438, USA; 2 Department of Discovery Chemistry, Merck Research Laboratories, West Point, Pennsylvania, 19438, USA

**Keywords:** Drug discovery, heteroaryldihydropyrimidines, phenylpropenamides, F-protein, monoclonal antibody, influenza, hepatitis B virus, human immunodeficiency virus, respiratory syncytial virus

## Abstract

Continued discovery and development of new antiviral medications are paramount for global human health, particularly as new pathogens emerge and old ones evolve to evade current therapeutic agents. Great success has been achieved in developing effective therapies to suppress human immunodeficiency virus (HIV) and hepatitis B virus (HBV); however, the therapies are not curative and therefore current efforts in HIV and HBV drug discovery are directed toward longer-acting therapies and/or developing new mechanisms of action that could potentially lead to cure, or eradication, of the virus. Recently, exciting early clinical data have been reported for novel antivirals targeting respiratory syncytial virus (RSV) and influenza (flu). Preclinical data suggest that these new approaches may be effective in treating high-risk patients afflicted with serious RSV or flu infections. In this review, we highlight new directions in antiviral approaches for HIV, HBV, and acute respiratory virus infections.

## Introduction

Viruses are intracellular pathogens that have evolved many devious strategies to evade host immune responses and, as a consequence, have plagued human health throughout history. Combating viral diseases with vaccines or antiviral drugs, or both, is a constant challenge. Even when successful strategies are discovered and employed, the high rate of genetic change exhibited by many viruses, particularly RNA viruses, often enables drug resistance or vaccine escape. This is compounded by the periodic emergence of new viral pathogens. Therefore, the continued search for new antiviral approaches is a noble cause that is critical for global human health.

Over the last several decades, significant resources in academic and biotechnological/pharmaceutical research have been directed toward chronic viral infections such as HIV, HBV, and hepatitis C virus (HCV), resulting in breakthrough therapies that have had a major impact on these chronic diseases. In fact, HCV drug development represents one of the greatest success stories in the history of antiviral therapy. Antiviral medications have been recently developed that can achieve a sustained viral response (SVR) (virus eradication or immune control of virus replication in the absence of continued therapy) in a majority of HCV-infected patients after short treatment durations ^1^. No less impressive has been the advent of combination antiretroviral therapy (cART) that has transformed HIV from a death sentence into a chronic but manageable condition. Similarly, some of the same approaches taken for HIV have been applied to HBV, resulting in the development of nucleoside/nucleotide analogues that effectively control viral replication and reduce the risk of HBV-associated disease, such as liver cirrhosis and hepatocellular carcinoma ^2^. Although current therapies for HIV and HBV are effective for controlling viral replication, they do not achieve virus eradication or SVR, as is the case for HCV therapies. Therefore, HIV and HBV treatment strategies continue to evolve.

Tremendous efforts are currently being applied to eradication, or cure, of HIV ^3^; however, this subject is beyond the scope of this review. In the absence of a viable cure for HIV, which even in the most optimistic view is still a decade away, the major unmet need in HIV treatment is to improve cART adherence. The prospect of lifelong therapy, combined with tolerability issues, leads to adherence challenges for many patients. In fact, more than 40% of patients with HIV experience some level of non-adherence over time on therapy ^4^, and this can lead to incomplete suppression of viral replication, emergence of drug resistance, and, ultimately, therapeutic failure. To address this issue, long-acting antiretroviral agents (e.g. cabotegravir and rilpivirine) are currently being advanced in the clinic with the hope that less frequent, or supervised, dosing may improve adherence to antiretroviral therapy or enable wider use of preventative treatment. Cabotegravir and rilpivirine target HIV integrase and reverse transcriptase, respectively, and are currently being studied in phase 3 clinical trials as a two-drug combination for HIV maintenance therapy. The cabotegravir and rilpivirine regimen is administered once every 4 or 8 weeks as multiple intramuscular injections following full suppression on an oral regimen ^5^. Recent data from the LATTE 2 trial showed that the injectable cabotegravir/rilpivirine combination maintained viral suppression rates comparable to a three-drug oral regimen of cabotegravir and two nucleoside reverse transcriptase inhibitors after 32 weeks ^6^. Patient surveys have indicated enthusiasm for long-acting parental therapies in the HIV community ^7^; however, it remains to be seen whether the reduction in dosing frequency and new administration route can improve adherence in certain patient populations. In addition, several questions remain about the potential liabilities of long-acting antiretrovirals, such as the risk of adverse events and drug resistance with prolonged exposure at subtherapeutic levels in patients who exhibit periodic lapses in adherence.

## Hepatitis B virus

HBV drug discovery efforts are currently geared toward increasing SVR rates, defined for HBV as HBsAg loss/seroconversion and control of HBV viral load in the absence of therapy. Current standard of care for chronically infected patients with HBV (CHB) is nucleoside/nucleotide analogue therapy (e.g. entecavir) or interferon (IFN). Nucleoside/nucleotide analogue therapy is highly effective at suppressing viral load, and a low percentage of treated CHB patients achieve SVR (<10%) after long-term treatment (2 to 4 years) ^2^. Similar rates of IFN-treated patients achieve SVR (<10%) after 48 weeks of treatment; however, IFN therapy is not well tolerated. Identification of direct-acting antivirals targeting new mechanisms in the HBV replication cycle that could be combined with nucleoside/nucleotide analogues or IFN therapy (or both) to achieve increased SVR rates is one concept under current investigation.

The HBV capsid represents an emerging HBV target that is potentially interesting. Originally, two classes of HBV capsid inhibitors were described: the heteroaryldihydropyrimidines (HAPs) and the phenylpropenamides ^8^. Mechanistic studies revealed that HAP compounds increase the kinetics of assembly and stabilize HBV capsid dimer interactions, resulting in aberrant capsid assembly ^9, 10^. Similar to HAPs, phenylpropenamides (e.g. AT-130) accelerate capsid assembly; however, this class of compounds also blocks RNA packaging, resulting in the formation of empty capsids rather than misdirecting capsid assembly ^11^. BAY 41-409, a member of the HAP class, demonstrated antiviral activity in an HBV transgenic mouse model and in humanized mice infected with HBV ^12, 13^. The disclosure of an HBV capsid/inhibitor X-ray cocrystal showed that the two classes of inhibitors shared overlapping binding sites on the HBV capsid ^14^. Recently, additional classes of HBV capsid inhibitors have been disclosed ^15, 16^, suggesting that the HBV capsid can be targeted by diverse chemical matter. In addition, capsid inhibitors with analogous activity have been identified for picornaviruses ^17^, HIV ^8^, and Dengue virus ^18^, strongly suggesting that viral capsid proteins may represent a viable target for a broad range of viruses. However, proof of concept (POC) in the clinic remains to be demonstrated.

## Respiratory syncytial virus and flu

In addition to chronic viral infections, a great deal of preclinical research has recently been focused on direct-acting antivirals against negative-stranded RNA viruses that cause respiratory infections, most notably for RSV and influenza (flu). Despite many years of intensive research efforts, there are very limited treatment options for these viral diseases that take their toll on the most sensitive of patient populations: the very young, the very old, and the immunocompromised. The development of efficacious flu vaccines has reduced morbidity and mortality in patients aged 6 months to 65 years, but inability to treat infants and reduced efficacy in the elderly leave a significant gap in patient coverage. Additionally, in years where strain mismatch occurs (e.g. 2014), a significant increase in the number of flu-related hospitalizations and deaths is observed ^19^. The currently available agents, which target either the neuraminidase enzyme or M2 protein, leave much to be desired in terms of efficacy, treatment window, and resistance profile. Our defense against RSV is even more sparse; there is no vaccine available, and ribavirin and palivizumab—Synagis (MedImmune, Gaithersburg, MD, USA), a monoclonal antibody (mAb) directed against the RSV fusion protein—are the only licensed agents for RSV; use of the former has been significantly reduced because of concerns over efficacy and toxicity, and the latter is approved only for prophylactic use in infants at highest risk.

The majority of reported drug discovery efforts against RSV over the past two decades have been focused on the F- (or fusion) protein, with multiple antibodies and small molecules, as well as a nanobody, entering development ^20^; some of these agents have been halted for efficacy, safety, or strategic reasons, but several are progressing through the clinic as highlighted below. An X-ray crystal structure of the post-fusion F-protein became available in 2011 ^21^, and it remains to be seen whether the structural insights gained have encouraged additional directed efforts at this validated target. Several other viral proteins, including the N-protein and the G-protein, have been targeted by antibodies, small molecules, and the first antiviral small interfering RNA (siRNA) ^20^. Inhibition of the L-protein, the RNA-dependent RNA-polymerase of RSV, seems an ideal target given its critical function in viral replication; however, little success has been realized prior to the 2014 disclosure of the leading L-protein inhibitor AL-8176 ( *vide infra*). The recent disclosure of an RSV replicon assay ^22^ and a screening cascade to identify RSV inhibitors in a target-agnostic fashion ^23^ demonstrate the continued desire both in academia and in the pharmaceutical industry to discover novel mechanisms of action for the treatment of RSV.

In terms of small-molecule development, 2014 was a watershed year that witnessed the first human POC for two mechanisms of action: the fusion inhibitor GS-5806 (Gilead, Foster City, CA, USA) ^24^ and the nucleoside inhibitor of the L-protein, AL-8176 (Alios, South San Francisco, CA, USA) ^25^ both demonstrated efficacy in human challenge studies ^26, 27^. The compounds were well tolerated, reduced symptoms associated with disease, and dramatically decreased viral load; the Alios nucleoside reduced viral burden in nasal washes to undetectable levels, whereas the fusion inhibitor produced a 4-log drop in viral titer. One is tempted to speculate that targeting the L-protein may lead to better efficacy (as suggested by undetectable viral load in the challenge study) and a higher barrier to resistance than targeting surface interactions; additionally, according to Alios pipeline reports, AL-8176 is also being advanced for additional paramyxovirus infections of medical concern: parainfluenza virus and human metapneumovirus ^28^. An agent such as this with broad-spectrum antiviral activity could be a true game-changer in the treatment of respiratory virus infections in high-risk populations. Other agents currently in clinical trials include second-generation (more potent and half-life extended) mAb against F-protein, MEDI-8897, (MedImmune); REGN-2222, another anti-RSV F mAb, (Regeneron, Tarrytown, NY, USA); an inhaled nanobody from Ablynx (Ghent/Zwijnaarde, Belgium), ALX-0171, directed against F-protein; and the inhaled siRNA from Alnylam (Cambridge, MA, USA), ALN-RSV01, that targets a conserved epitope on N-protein. Additionally, the F-protein inhibitor AK0529, from Ark Biosciences (Shanghai, China), recently completed phase 1 studies and is now undergoing phase 2 evaluation in hospitalized infants ^29^. Given the high unmet medical need and several tractable, validated mechanisms to target, we expect to see a continued high level of competition in the RSV space and are confident that novel agents will begin to arrive on the market in the near future.

Flu research has witnessed a recent surge in activity, and most efforts are focused on novel mechanisms of action that may have potential for improved efficacy, treatment window, and resistance profile when compared with neuraminidase or M2 protein inhibitors. To this end, we feel that the brightest future lies in inhibition of one or more components of the viral polymerase complex. Unlike most negative-stranded RNA viruses, Orthomyxoviruses such as flu use a unique heterotrimeric polymerase complex composed of the PA protein (endonuclease), the PB1 protein (the RNA-dependent RNA-polymerase, or RdRp), and the PB2 protein (cap-snatching subunit) that work together in a tightly associated and coupled fashion ^30^. The past decade has seen significant advances in our understanding of the structure and function of the subunits of the polymerase complex, culminating in the recent elucidation of the full heterotrimeric polymerase complex of flu A ^31^, flu B ^32^, and flu C ^33^ by X-ray crystallography. As is the case for targeting RSV polymerase, the flu polymerase complex is a compelling target for novel antivirals because of its role in not just reducing the spread of infection but also halting all intracellular replication; this may lead to an expanded window for intervention and reduced likelihood for the generation of drug-resistant viruses. Compounds targeting each of the three components of the polymerase have now reached the stage of clinical evaluation.

The most advanced compound is the PB1 inhibitor Favipiravir (T-705), which is approved for use against pandemic flu in Japan; additional clinical studies are ongoing around the world to study its safety and efficacy in the treatment of uncomplicated flu. The pyrazinecarboxamide of T-705 is converted to a nucleoside analog *in vivo* and is believed to act via incorporation into viral RNA by the PB1 RdRp; however, as with ribavirin, it is a non-specific nucleoside that is active against multiple viruses and has been shown to induce lethal mutagenesis in flu strains *in vitro* ^34^. Favipiravir has also shown efficacy for Ebola infection in mouse models of disease ^35, 36^ and was administered post-infection during the recent Ebola outbreak, where it showed some evidence of efficacy if administered prior to significant disease onset ^37^. Development of nucleosides that specifically target the PB1 protein has been a substantial challenge in the field; however, a recent patent application from Riboscience (Palo Alto, CA, USA) suggests that potent and specific nucleosides for this purpose may be achievable ^38^. The current stage of development of these nucleoside analogs is unknown.

The most exciting development of the past several years has been identification of the first inhibitor of the cap-snatching function of the PB2 protein via a phenotypic screening approach ^39^. In 2013, Vertex (Boston, MA, USA) achieved POC in a human challenge study with their leading PB2 inhibitor, VX-787, wherein they demonstrated significant reductions in viral load and symptom duration when drug was administered, beginning 24 hours after exposure to the virus, once a day for 5 days ^40^. Preclinical data from a lethal mouse flu model indicate that VX-787 does indeed expand the treatment window to 96 hours post-infection; in contrast, oseltamivir must be given within 48 hours to provide a measure of protection in this model ^41^; however, delayed treatment efficacy has not yet been tested in humans. In June 2014, Janssen (Beerse, Belgium) entered into a collaborative agreement with Vertex to develop and commercialize the renamed compound (JNJ-872). JNJ-872 has broad-spectrum activity against flu A strains but does not have activity against flu B. This observation can be readily explained by examination of the X-ray structure of JNJ-872 bound to the flu A PB2 protein, wherein many of the amino acids in direct contact with the inhibitor are not conserved between the major flu A and B strains ^41^. These structural data suggest that identification of a second-generation inhibitor that provides efficacy against both flu A and B will be very challenging to obtain within a similar binding motif. Roche (Basel, Switzerland) and a small biotech, Savira (Vienna, Austria), founded by the group that solved the full flu polymerase structures, partnered in 2013 to develop flu polymerase inhibitors, but their current stage of development is unknown.

The endonuclease function of the PA protein is the subunit with the longest history; the first inhibitors were reported by Merck (Kenilworth, NJ, USA) in the mid-1990s ^42^. This enzyme uses a similar active site as HIV integrase; in fact, it was these early endonuclease inhibitors that led Merck to identify, via high-throughput screening of their sample collection, the key pharmacophore that has supplied all of the chemical matter advanced to date for the integrase enzyme, including the three US Food and Drug Administration-approved integrase inhibitors. The challenging pharmaceutical properties of small molecules required to inhibit endonuclease has likely slowed progress, but advances in HIV integrase compound design, as well as X-ray elucidation of the PA subunit in complex with inhibitors ^43, 44^, may have informed researchers how to make more drug-like endonuclease inhibitors. In fact, two companies have endonuclease inhibitors in clinical stage evaluation: Shionogi (Osaka, Japan) has demonstrated safety and acceptable pharmacokinetics of S-033188 in a phase 1 study in Japan ^45^, and Janssen (through their acquisition of AL-794 from Alios) has an endonuclease inhibitor currently in phase 1 clinical studies in healthy volunteers in the US ^46^. Though there is not yet POC for this mechanism, endonuclease’s vital function in the polymerase complex makes its clinical success highly probable. Furthermore, there is very high homology between flu A and B in the endonuclease active site, suggesting that this could be a better target than the currently identified site on PB2 to obtain broad-spectrum flu activity. If Janssen’s endonuclease compound AL-794 advances, they could be in a position to test combinations with JNJ-872 as early as 2017, providing the first look at the potential synergy in combining the inhibition of two separate components of the polymerase complex.

Utilization of broadly neutralizing mAbs targeting the Flu hemagglutinin (HA) protein represents a separate approach to develop flu therapeutics. With advances in technologies to isolate antibodies directly from human B cells, many anti-flu HA mAbs with broad neutralizing activity have been identified ^47– 54^. Several such mAbs—FI6, CR9114, and 39.29 (MHAA4549A)—that target the highly conserved stem region of HA demonstrate potent activity against all, or nearly all, flu A strains tested ^51, 53, 54^. One potential indication of interest is the utilization of flu mAbs to treat patients hospitalized with severe flu infection. Preclinical animal models indicate that flu mAbs exhibit superior efficacy compared with oseltamivir in mouse or ferret models of lethal flu infection when treatment is initiated later in infection ^54^. Multiple mechanisms of antiviral activity have been described for flu mAbs, including Fc effector function-mediated activity ^55^, providing some rationale for the improved activity observed in preclinical models. MHAA4549A is currently being developed in the clinic and has demonstrated POC in a human challenge study; however, the dose required for efficacy was high (3.6 grams). Despite the high dose requirements, MHAA4549A is progressing to phase 2 trials in hospitalized patients and will test the utility of HA stem-binding mAbs in this at-risk population. A second HA stem-binding mAb currently being developed by Visterra (Cambridge, MA, USA), VIS410, also recently demonstrated POC in a human challenge study at a similar high dose (2.3 grams) ^56^.

## Conclusion

Though some pharmaceutical companies have recently announced a reduction or elimination of their efforts toward antiviral drug discovery, the future of research in this area remains bright; several large companies, as well as many biotechnology companies and academic researchers, are pushing the frontiers of knowledge and unearthing new approaches to combating viral infections. Along with the exciting future in new treatments for HIV, HBV, RSV, and flu highlighted above, nascent reports suggest that tractable approaches to treat viral diseases such as norovirus, Dengue, and Ebola are also within reach. Furthermore, the largely unexplored and poorly understood area of targeting the host immune system and harnessing its power to clear viral infections is gaining traction, and we are hopeful that such an approach may one day allow the discovery of heretofore unknown single agents that have the potential to cure diseases caused by a broad range of genetically distinct viruses ^57^.

## Abbreviations

cART, combination antiretroviral therapy; CHB, chronic hepatitis B; flu, influenza; HA, hemagglutinin; HAP, heteroaryldihydropyrimidine; HBV, hepatitis B virus; HCV, hepatitis C virus; HIV, human immunodeficiency virus; IFN, interferon; mAb, monoclonal antibody; POC, proof of concept; RdRp, RNA-dependent RNA-polymerase; RSV, respiratory syncytial virus; siRNA, small interfering RNA; SVR, sustained virological response.
